# Primary Central Nervous System Lymphoma in an Immunocompetent Patient Presenting as Multiple Cerebellar Lesions: A Case Report and Review of Literature

**DOI:** 10.1177/2324709619893548

**Published:** 2019-12-10

**Authors:** Gliceida M. Galarza Fortuna, Kathrin Dvir, Christopher Febres-Aldana, Michael Schwartz, Ana Maria Medina

**Affiliations:** 1 Mount Sinai Medical Center, Miami Beach, FL, USA; 2 Mount Sinai Comprehensive Cancer Center, Miami Beach, FL, USA; 3 Florida International University, Miami, FL, USA

**Keywords:** primary CNS lymphoma, lymphoma, CD10, brain tumor, cerebellar tumor

## Abstract

Primary central nervous system (CNS) lymphoma (PCNSL) is an uncommon extranodal non-Hodgkin lymphoma often presenting as a single brain lesion within the CNS. On histopathological evaluation of PCNSL a positive CD10, which is frequently observed in systemic diffuse large B-cell lymphoma, is present in approximately 10% of PCNSL. We describe a case of CD10-positive PCNSL presenting with multiple posterior fossa enhancing lesions in an immunocompetent older woman with a history of breast cancer successfully treated by the RTOG 0227 protocol consisting of pre-irradiation chemotherapy with high-dose methotrexate, rituximab, and temozolomide for 6 cycles, followed by low-dose whole-brain radiation and post-irradiation temozolomide.

## Background

Primary central nervous system (CNS) lymphoma (PCNSL) is an uncommon extranodal non-Hodgkin lymphoma. This entity often involves the brain, leptomeninges, vitreoretinal compartment, or spinal cord, without systemic involvement. Histologically, this malignancy is commonly classified as diffuse large B-cell lymphoma (DLBCL).^1-3^ PCNSL often presents as a single brain lesion within the CNS, with a supratentorial location, in approximately 60% of patients.^4^ We describe a case of PCNSL presenting with multiple posterior fossa enhancing lesions in an immunocompetent older woman with a history of breast cancer.

## Case Presentation

A 78-year-old Caucasian woman of Jewish Ashkenazi descent visited her primary care physician for progressive right-sided ataxia of 1 week. Noncontrast head computed tomography (CT) showed a focal area of decreased attenuation within the right cerebellar hemisphere extending into the right cerebellar peduncle with associated vasogenic edema. The patient had a history of estrogen and progesterone receptors positive, right breast ductal cell carcinoma in situ, which was fully excised. Initially the brain imaging findings were suspected to be metastatic disease and the patient was treated with a 3-day course of steroids before being transferred to our institution for further workup and treatment.

**Figure 1.**
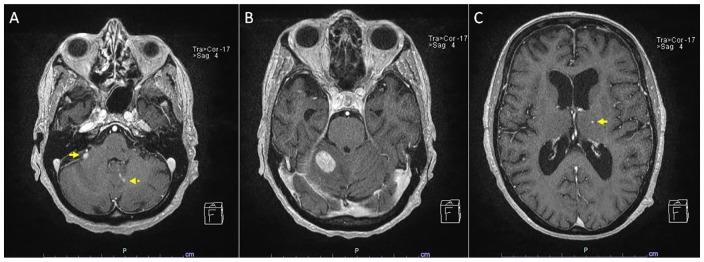
Brain MRI showing multiple enhancing brain lesions including a 0.7-cm right superior anterior cerebellar parenchymal lesion (A, arrow), 0.3-cm and 0.2-cm left paramedian cerebellar vermian lesions (A, dashed arrow), a 1.9-cm superior right cerebellar lesion with mass effect and surrounding vasogenic edema causing partial effacement of the right aspect of the fourth ventricle (B), and a 0.2-cm left thalamic lesion (C, arrow).

On admission to our institution, repeat noncontrast head CT showed an iso-hypodense ill-defined lesion in the right cerebellum measuring approximately 1.6 cm associated with surrounding edema and mild mass effect on the fourth ventricle. A follow-up brain magnetic resonance imaging (MRI) showed multiple posterior fossa enhancing lesions and an additional punctate enhancing lesion in the left thalamus, suspicious for metastatic carcinoma (Figure 1). MRI of the cervical, thoracic, and lumbar spine was unremarkable. CT scan of the chest, abdomen, and pelvis showed no evidence of metastatic disease or primary tumor in these regions. No lumbar puncture was ever performed as was deemed contraindicated by the neurosurgical team.

**Figure 2.**
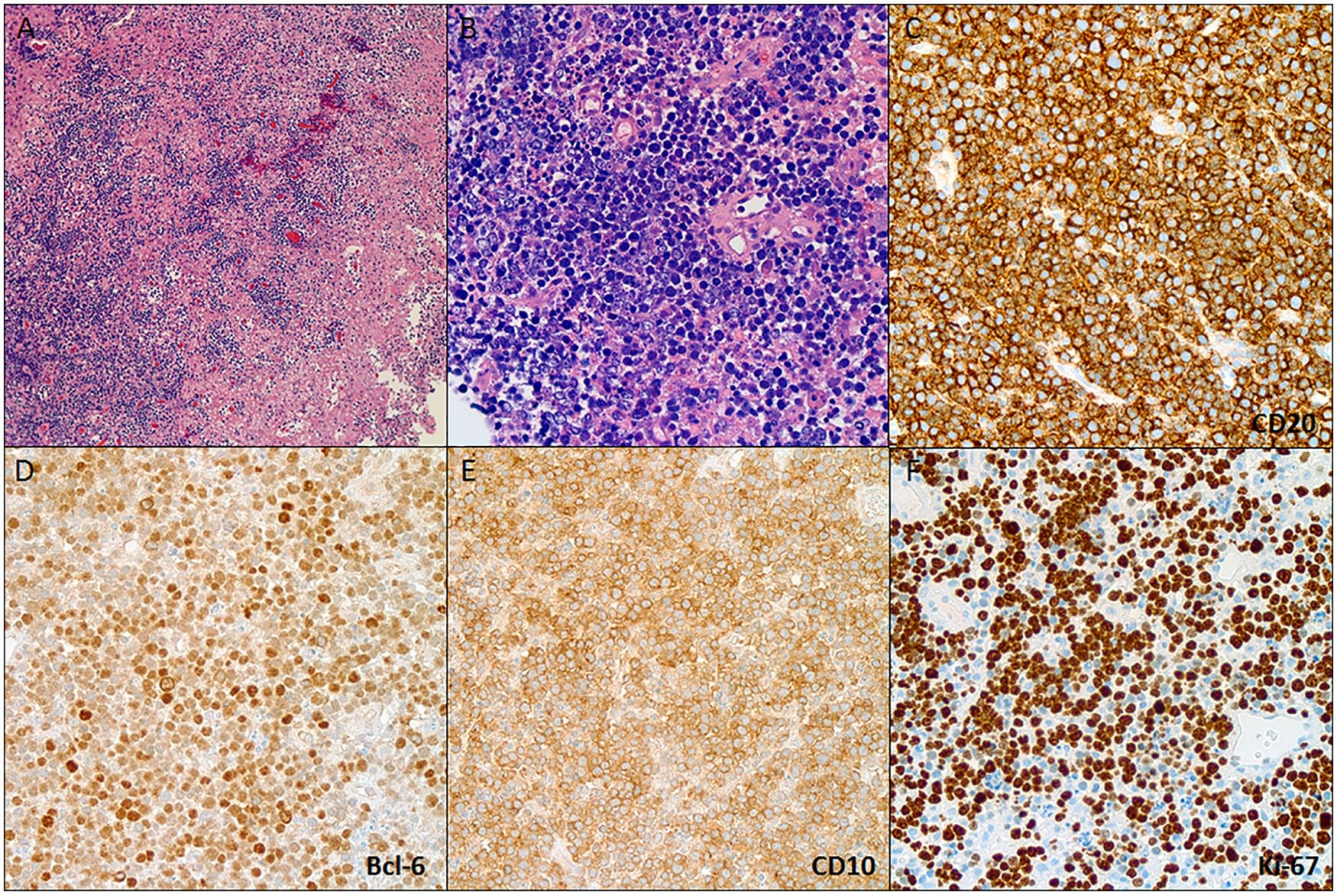
Brain biopsy showing a cellular infiltrate with necrosis and perivascular preservation (A) composed of large lymphoid cells with centralistic morphology and numerous apoptotic bodies (B), immunoreactive for CD-20 (C), Bcl-6 (D), and CD-10 (E) consistent with a DLBCL of germinal center phenotype. The lymphoma exhibited a high proliferation index (>95%).

Consequently, the patient underwent a right-sided posterior fossa craniotomy with excisional biopsy of the right cerebellar lesion, using 3-dimensional navigational guidance. Histopathology of the specimen showed a DLBCL positive for CD20 and CD10 with a Ki67 proliferative index greater than 95% (Figure 2). Epstein-Barr virus analysis by EBER in situ hybridization was negative. Fluorescence in situ hybridization for *t*(14;18) and rearrangement of *BCL6* and *MYC* genes were negative. Bone marrow biopsy showed no evidence of lymphoma. The patient was managed as per the RTOG 0227 protocol of pre-irradiation chemotherapy with high-dose methotrexate, rituximab, and temozolomide for 6 cycles, followed by low-dose whole-brain radiation and post-irradiation temozolomide.

**Figure 3.**
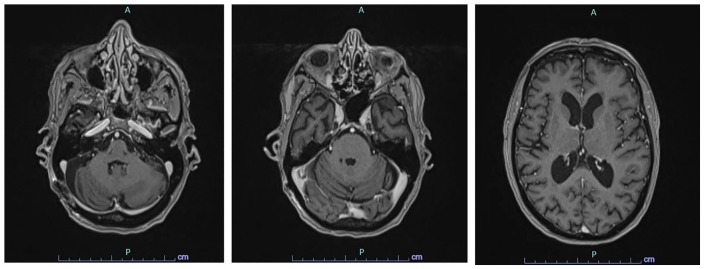
Brain MRI after 6 cycles of chemotherapy completion showing interval resolution of the multiple enhancing lesions in the posterior fossa. No evidence of residual or new foci of primary CNS lymphoma.

At the time of this report, the patient had completed chemotherapy and whole-brain radiation, which she tolerated well. No ataxia or other focal neurological deficits were noted on her last physical examination. A follow-up brain MRI showed interval resolution of the multiple enhancing lesions in the posterior fossa, with no evidence of residual or new foci of primary CNS lymphoma (Figure 3).

## Discussion

PCNSL represents approximately 4% of all intracranial malignancies, 5% of all extranodal lymphomas, and less than 1% of all non-Hodgkin lymphomas.^4^ The most common histologic presentation is DLBCL, representing approximately 95% of PCNSL. Less common are T-cell lymphoma, Burkitt’s lymphoma, lymphoblastic lymphoma, and marginal zone lymphoma.^3^

PCNSL is mainly reported in immunosuppressed patients, with HIV/AIDS being among the most important risk factors. Twenty-six percent of all PCNSL cases in the United States between the years 1980 and 2007 were diagnosed in HIV-positive individuals. However, most recent studies show a significant decrease in PCNSL cases among HIV patients, which has been attributed to the widespread utilization of antiretroviral therapy.^5,6^ Conversely, the incidence of PCNSL in immunocompetent hosts continues to increase, mostly in the elderly population.^1,6^ Recent epidemiological studies have shown a higher incidence of PCNSL in immunocompetent, males and individuals more than 75 years old. African Americans tend to present at a younger age, of less than 50 years old, while Whites present older than 50 years old.^1^ Moreover, a relationship between breast cancer and the development of non-Hodgkin’s lymphoma has been described in the literature.^7^ The lymphoma diagnosis often follows the breast cancer diagnosis and is not induced by chemotherapy or radiation offered as breast cancer treatment.^7,8^

The clinical presentation of PCNSL varies greatly, depending mainly on the primary location of the lesion(s). The symptoms often progress rapidly and are nonspecific. Approximately 50% of the patients present with focal neurologic findings, while the other 50% present with neuropsychiatric changes. Signs and symptoms associated with increased intracranial pressure, such as nausea, headache, and seizures, are not often seen.^9^

PCNSL continues to be a diagnostic challenge, given that its radiographic features are commonly seen in other CNS conditions. Therefore, a pathological examination is mandatory to make a PCNSL diagnosis. There are some situations where the pathologic assessment of PCNSL can be compromised. Patients with intracranial space-occupying lesions are often treated with corticosteroids, to which lymphomas are sensitive. Corticosteroid pretreatment often leads to apoptosis and morphologic changes of the lymphoma cells, which can decrease the diagnostic yield of tissue biopsy.^2,10^ Pathological evaluation of corticosteroid pretreated PCNSL leads to a final diagnosis in only 50% to 85% of the cases.^9^ The sensitivity is also notably reduced in cases when tissue is obtained through a stereotactic biopsy, which is the preferred method for sampling brain tissue.^2,11^

Brain imaging is commonly included in the workup of patients with focal neurologic deficits, or other symptoms that could be attributed to intracranial space-occupying lesions. On CT scan, PCNSL in immunocompetent individuals often presents as a single hyper- or iso-attenuated lesion. Likewise, on MRI, these lesions tend to be hypo- or iso-intense in unenhanced T1-weighed sequencing and hyper- or iso-intense on unenhanced T2-weighted images. Most PCNSL have significant contrast uniform enhancement with or without necrosis on CT and MRI.^11^ These characteristics, however, are not diagnostic of PCNSL as many other processes share the same characteristics: metastatic lesions, meningiomas, malignant gliomas, abscess, multiple sclerosis, and others.^12^

PCNSL usually presents as a single focal lesion. However, approximately 18% to 30% of the cases of non-HIV PCNSL had multiple lesions.^11,12^ These lesions are often supratentorial (87%), with frequent involvement of the frontoparietal lobes (39%).^4^ Cerebellar involvement has been described in approximately 9% to 25% of newly diagnosed PCNSL.^11,13^ Our patient’s case highlights 2 unusual radiographic appearances of non-HIV PCNSL, which are multiple enhancing lesions and a posterior fossa location.

Pathologic evaluation of PCNSL commonly reveals DLBCL characterized by sheets of large lymphoma cells that are highly proliferative (demonstrated by high Ki-67 expression) with large areas of necrosis that may harbor viable perivascular lymphoma islands.^14^ PCNSL in non-HIV patients does not tend to show the presence of Epstein Barr virus or histological changes related to this viral infection.^15^ The tumor cells in primary CNS DLBCL are positive for B-cell markers (Pax5, CD19, CD20, CD79a) with either kappa or lambda light chain restriction. CD10 is positive in less than 10% of these lymphomas. CD10 expression is more frequent in systemic DLBCL; therefore, CD10 positivity in a CNS DLBCL should prompt a search for systemic DLBCL that has disseminated to the CNS.^14^ DLBCL in other locations is often subdivided in germinal center B-cell (GCB) or non-germinal center B-cell subtypes, according to expression of CD10, BCL-6, and MUM1.^16,17^ The GCB phenotype is associated with a better outcome when compared with the non-GCB subtype.^18^ However, this association has not been confirmed in PCNSL. Despite CD10 positivity, systemic DLBCL was ruled out in this patient; hence, this case is an example of an unusual CD10+ primary CNS DLBCL. Additionally, as often seen in PCNSL, the sample showed a high Ki-67 proliferation index (greater than 95%), which has been associated with aggressive lymphomas and thus, entails poor prognosis.^19^

PCNSL is known to have a very aggressive course, with a high recurrence rate following treatment. The current management of PCNSL consists of induction and consolidation therapies. Induction therapy consists of a combined poly-chemotherapy with high-dose methotrexate, an alkylating agent and rituximab. The overall survival at 10 years after high-dose methotrexate-based therapy is 35%.^4^ If a good response to the combined therapy is achieved, it is followed by consolidation therapy with either whole-brain radiotherapy or autologous stem-cell transplantation.^13^ However, approximately half of the patients with initial response, relapse, with median overall survival in relapsed patients varies between 41 and 62 months.^4,20^ Although surgical resection is not the standard of care, surgery might be considered if symptoms developed rapidly, and if the patients develop signs and symptoms compatible with brain herniation.^20^ If no treatment is pursued, PCNSL survival has been estimated to be 3.3 months.^21^

## Conclusion

Primary CNS lymphomas are usually DLBCL, which are inherently aggressive brain tumors. This entity has been linked with immunosuppressive states, namely, HIV/AIDS. However, the incidence in immunocompetent elderly patients, mostly men, is increasing.

In this case, we report of a 78-year-old female patient with a history of breast carcinoma, who presented with new onset of unilateral ataxia and a brain MRI showing posterior fossa and thalamic enhancing lesions, highly suspicious for metastatic carcinoma. A primary CNS process was not initially suspected and the diagnosis was made possible solely as a result of histological examination. This case emphasis the importance of biopsy with a histopathological analysis for an accurate diagnosis and management of brain tumors and that a metastatic disease should not be always assumed.
